# Process Engineering Strategies for Microbial Lipid Production: From Strain Evolution to Industrial-Scale Bioprocessing

**DOI:** 10.3390/ijms27093760

**Published:** 2026-04-23

**Authors:** Eusebiu Cristian Florea, Adelina Gabriela Niculescu, Andreea Gabriela Bratu, Dan Eduard Mihaiescu, Alexandru Mihai Grumezescu

**Affiliations:** 1 Research Institute of the University of Bucharest—ICUB, University of Bucharest, 050657 Bucharest, Romania; eusebiu.florea@stud.chimie.upb.ro (E.C.F.); adelina.niculescu@upb.ro (A.G.N.); 2 Faculty of Chemical Engineering and Biotechnologies, National University of Science and Technology Politehnica Bucharest, Gh. Polizu St. 1–7, 060042 Bucharest, Romania; andreea.bratu2910@stud.fim.upb.ro (A.G.B.); dan.mihaiescu@upb.ro (D.E.M.)

**Keywords:** microbial oil, microbial lipids, fermentation, adaptive laboratory evolution, feedstocks, cell disruption, biomass

## Abstract

Microbial lipids have emerged as a promising sustainable alternative to plant- and petroleum-derived oils, with applications spanning biofuels, oleochemicals, nutraceuticals, and specialty materials. Significant advances in metabolic engineering and strain development have increased lipid production capacity across diverse microorganisms. Numerous reviews have summarized the biological and metabolic advances in this field, highlighting significant progress in metabolic engineering and strain development that has increased lipid production capacity across diverse microorganisms. However, translating these gains into economically viable industrial processes remains a major challenge. This review examines process engineering strategies for microbial lipid production across the full bioprocessing pipeline, from laboratory-scale strain evolution to industrial-scale operation. We discuss recent developments in adaptive laboratory evolution, systems-guided strain optimization, and robustness engineering, emphasizing their implications for process performance. Key bioprocess parameters—including substrate selection, nutrient limitation strategies, reactor design, oxygen transfer, and process control—are critically evaluated for their impact on lipid yield, productivity, and scalability. Furthermore, downstream processing considerations and techno-economic constraints are analyzed in the context of large-scale implementation. By integrating strain-level innovations with process engineering principles, this review highlights current bottlenecks, emerging solutions, and future directions for achieving efficient and scalable microbial lipid biomanufacturing.

## 1. Introduction

Microbial oil is a specific type of storage lipid produced by microorganisms. It is produced by oleaginous microbes that accumulate 20% to 80% lipids per dry biomass during the stationary growth phase under nutritional restrictions, such as nitrogen or phosphorus, alongside carbohydrates, hydrocarbons, and fats as carbon sources [1,2,3,4]. These lipid reserves are composed of triacylglycerols (TAGs) containing long-chain fatty acids and sterol esters, which are analogous to those found in edible lipids and oils derived from both animal and plant sources. Significant quantities of other lipid components may include free fatty acids (FFAs), neutral lipids, sterols, and polar fractions, including phospholipids, sphingolipids, and glycolipids [4,5,6]. Biodiesel is routinely produced by transesterification of TAGs, yielding fatty acid methyl esters (FAMEs) and fatty acid ethyl esters (FAEEs). The best candidates for TAG production are microalgae, bacteria, molds, and yeast [7,8]. Triglycerides composed of polyunsaturated fatty acids (PUFA) have been identified as the main components of microbial oil. In contrast, the fatty acids with high unsaturation degree (e.g., C16 and C18) are chemically similar to vegetable oils (e.g., rapeseed oil, palm oil, soybean oil) [1].

Single-cell oils (SCOs) offer considerable sustainability potential by enabling production from alternative feedstocks, such as agricultural waste, thereby diminishing dependence on land-intensive crops. They offer benefits over conventional vegetable and fossil fuels, including reduced reliance on seasonal and geographical constraints, higher growth rates, and greater resource efficiency. Nonetheless, obstacles persist, such as the need to optimize processes to enhance cost-effectiveness and efficiency, increase production capacity, and ensure food safety [9,10]. The environmental impacts of SCO production are markedly beneficial, as these microbes can thrive in non-agricultural soils and utilize waste streams, thereby preserving land and water resources while reducing greenhouse gas emissions. Moreover, microbial oils may be modified to yield oils with preferred characteristics and customized fatty acid compositions, hence improving their nutritional benefits and functional uses. Transitioning to microbial oil production can fulfill the urgent demand for sustainable, resource-efficient, and ecologically benign edible oil alternatives [3].

The use of filamentous fungi as SCO producers has the potential to integrate saccharification and fermentation, thereby optimizing the use of inexpensive substrates, including agro-industrial waste and lignocellulosic materials, and enhancing the cost-effectiveness of the process [11].

Although numerous studies have investigated the genetic and metabolic engineering of microbial strains for lipid production, as well as isolated aspects of bioprocess optimization, there is a lack of integrative reviews that link laboratory-scale strain evolution with process engineering strategies for industrial-scale lipid biomanufacturing [6,12]. In this context, this review fills the gap by offering a thorough, process-centric perspective that connects evolutionary strain development, bioprocess design, and scale-up engineering concepts to facilitate the transition of microbial lipid synthesis from the laboratory to industrial application.

## 2. Engineering Considerations in Microbial Lipid Production

Certain metabolic pathways that reroute carbon flux toward fatty acid and TAG production control microbial lipid accumulation. Glycolytic flow produces pyruvate under carbon surplus and nutritional constraints (usually nitrogen), which is then transformed into mitochondrial acetyl-CoA and citrate. Citrate builds up and is exported to the cytosol, where it is used by adenosine triphosphate (ATP)-citrate lyase to regenerate acetyl-Coenzyme A (CoA), which is the building block for fatty acid production. After being carboxylated to malonyl-CoA, acetyl-CoA is lengthened by fatty acid synthase complexes to create long-chain fatty acids, which are then esterified with glycerol-3-phosphate to create TAGs. These lipids are kept as energy stores inside the cell. As will be covered in later sections, this metabolic change explains why lipid formation is highly dependent on the C/N ratio and nutritional constraints [13,14,15].

Microbially synthesized oil has become a viable option for the sustainable production of biodiesel amid increasing environmental concerns. However, high production costs limit its widespread use. Consequently, it is essential to engineer bioreactor cultivations with minimal operational demands and elevated yields, tailored to an oleaginous system [16,17]. The operational modes of bioreactors (batch, fed-batch, continuous, and two-stage systems) exhibit considerable variation in their management dynamics and productivity, which directly influences the yield of yeast-derived biomass and lipids [18].

To shed some light on the engineering considerations in microbial lipid production, the following subsections provide an overview of bioreactor configurations and key process parameters through both descriptive and comparative discussions.

### 2.1. Bioreactor Configurations Used for Microbial Lipid Production

#### 2.1.1. Batch Systems

The standard batch procedure is the simplest method for microbial synthesis of chemicals or bioremediation. Batch production involves cultivating cells in a vessel with a predetermined concentration of feed supply and culture medium. No additional nutrients are introduced or removed from the system. As the amount of biomass within the tank increases, the beginning feed source is progressively depleted, while metabolic debris and products accumulate in the medium. Bioreactor fermentation is used to determine optimal operating parameters for lipid production, including pH, agitation rate, aeration rate, and initial media composition. The Carbon-to-Nitrogen (C/N) ratio can be adjusted through ammonia addition for pH management, as ammonia not only modifies pH but also supplies nitrogen, which affects microbial nitrogen utilization and process stability. To date, slightly acidic environment conditions (pH 5.5–7) have been demonstrated to be beneficial to lipid synthesis in oleaginous yeasts. However, Gao et al. [19] indicated that alkaline conditions (optimal pH 8) effectively reduced the detrimental impacts of increased volatile fatty acids (VFAs) concentrations on oleaginous yeast, resulting in a substantial enhancement of biomass output and lipid production.

Large-scale synthesis of commodity chemicals rarely uses standard batch procedures because of productivity losses during reactor cleaning and the need to regenerate cells after each fermentation cycle. Although this type of cultivation has limitations, it is a straightforward method that does not involve complex control loops to manage subsequent nutrition or waste removal [20,21]. The original C/N ratio of the culture medium is crucial in determining the performance of batch cultures in bioprocesses. Higher levels of lipid-free biomass generated during the growth phase at a fixed carbon concentration correlate with higher lipid production rates during the lipogenic phase. The oleaginous yeasts will continue to grow until nitrogen is depleted, resulting in a gradual increase in the residual C/N ratio until carbon is completely utilized [22,23].

#### 2.1.2. Fed-Batch Systems

**Figure 1:**
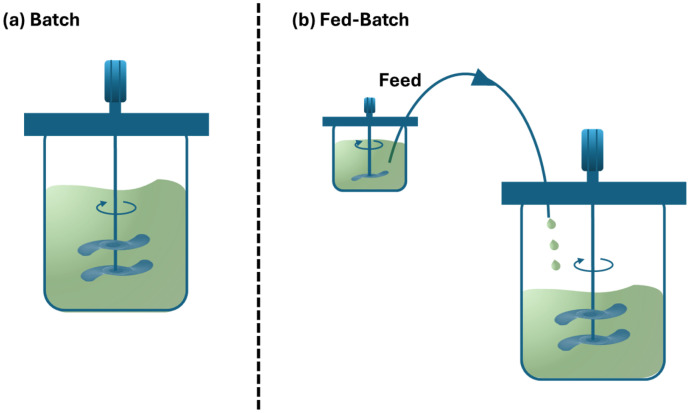
(**a**) Batch and (**b**) fed-batch bioreactor processes. Created based on information from [21,30].

Fed-batch culture has traditionally been regarded as an efficient method for enhancing cell densities. In this setup, nutrients are supplied continuously or intermittently (bolus) to the system to boost reactor contents and regulate overall substrate concentrations. This mode is widely used in industry for its effectiveness in producing non-growth-associated products and as a robust alternative to complex continuous feeding protocols. Moreover, it regulates substrate concentrations, as elevated levels may be inhibitory or alter metabolic pathways. It maintains substrate concentration within an optimal range, preventing microorganism inhibition. It is essential to allocate space within the system to facilitate medium insertion. The use of orange peel as a substrate for lipid production by oleaginous yeasts represents a promising strategy to enhance the development of more sustainable and circular bioeconomic models [21,24,25,26]. By the end of the fermentation process, a significant amount of carbon is transformed into cells and lipids, demonstrating that initial substrate inhibition can be avoided. The incorporation of medium diluents reduces cell concentration; thus, fermentation can be classified into two categories: fixed-volume (when broth is extracted and an equivalent volume of feed solution is introduced) or variable-volume, as observed in constant-rate feeding scenarios [27,28]. Using a constant substrate concentration technique maintains a steady growth rate, leading to an exponential increase in the cell population in the bioreactor over time. Nonetheless, this implies that the system needs to be fed exponentially with substrates. Industries frequently employ periodic feeding (bolus) due to its simplicity and high efficiency. The batch and fed-batch modes are illustrated in Figure 1 [24,29].

Fed-batch fermentation effectively regulates the C/N ratio more efficiently than conventional batch culture, facilitating precise control to enhance growth, avoid inhibition, and steer metabolism towards targeted products (such as lipids or proteins) by preventing nutrient depletion or surplus, which is essential for optimizing yield and productivity [31,32].

#### 2.1.3. Continuous Systems

**Figure 2:**
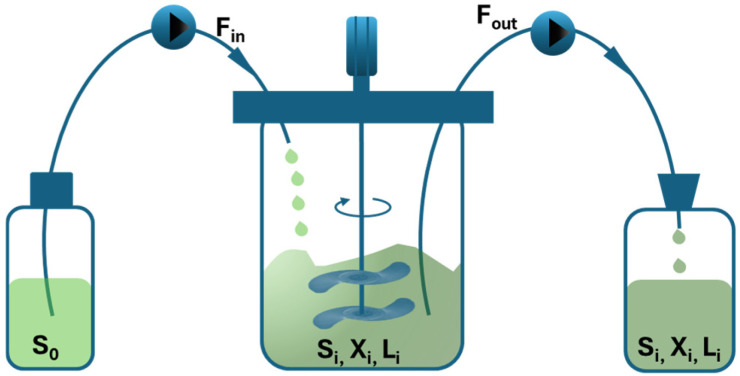
Schematic depiction of continuous cultivation. Created based on information from [16,28].

Both carbon and nitrogen inputs are necessary for continuous functioning, which can sustain a steady state C/N ratio. A schematic representation of continuous culture is shown in Figure 2, where S_0_ denotes the concentration of substrate in the feed medium, while X_i_, S_i_, and L_i_ represent the concentrations of cells, substrate, and lipids in the broth and bleed collection bottle, respectively. F_in_ signifies the flow rate of the feed to the bioreactor, while F_out_ indicates the flow rate of the bleed from the bioreactor.

By adjusting the residual nitrogen concentration, it is possible to target either proliferation or fat accumulation while maintaining a constant growth rate, cell concentration, and substrate absorption rate. Continuous fermentation outperforms batch mode in terms of economic feasibility and lipid productivity, as cells can be continuously harvested and processed. Continuous delivery of nutrients prevents nutrient restriction from excessive substrate, ensuring that cells are provided with a new nutrient medium, similar to fed-batch mode with ongoing feed supply. The principal determinant of efficiency in a continuous system is the dilution rate [16,23]. Dilution rates below 0.06 h^−1^ have been demonstrated to optimize lipid synthesis in continuous cultures of various yeasts [33].

Lipid production can be performed in a large continuous batch reactor volume (1000 L). Lipid accumulation in planktonic bacteria has been examined at a comparable scale, yielding analogous titer (about 6 g/L) and productivity (approximately 130 mg/L/h) as microalgae and yeast [34]. Continuous operation maximizes productivity by maintaining a constant C/N ratio and steady production yields, while also reducing the time required for cleaning, sterilization, and handling of the vessel. However, the process requires optimization to achieve the optimal N level, making it difficult to neatly separate products into batches for traceability while also increasing the risk of strain oil degradation, contamination, and blockages [16,35].

#### 2.1.4. Two-Stage Systems

**Figure 3:**
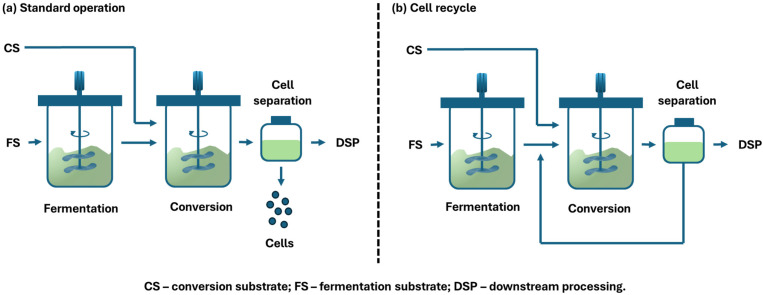
Schematic depiction of -TSF systems in (**a**) conventional operation and (**b**) with cell recycling. Created based on information from [38,40].

The two-stage fermentation (TSF) procedure separates lipid accumulation and cell proliferation phases, thereby optimizing conditions for both culture stages [36]. A two-stage continuous system, typically represented by two continuously stirred tank reactors in series, facilitates spatial segregation of microbial processes to improve productivity. This procedure is typically characterized by an initial growth phase focused on biomass accumulation, followed by a second phase that functions under nutrient constraints or stress conditions to optimize product synthesis [37]. Figure 3 depicts TSF as a process schematic, encompassing a decoupled fermentation phase and a conversion phase, potentially using a conversion substrate in conventional operation and with cell recycling. In the standard operation, cells are separated, and the product stream proceeds to downstream processing. The removed cells are either discarded (Figure 3a) or recycled back to the conversion phase (Figure 3b). The initial fermentation stage, characterized by a low C/N ratio and abundant nutrients, facilitates rapid growth and yeast cell reproduction, whereas the subsequent fermentation stage, marked by a high C/N ratio and nitrogen limitation, prompts cells to rapidly accumulate lipids. A significant benefit of fermenting predominantly during the production phase is reduced substrate consumption for excess biomass production. Limiting excessive biomass growth can also benefit other cost factors. Elevated cell density also increases viscosity, thereby reducing the oxygen exchange rate, potentially leading to additional costs for supplying pure oxygen to the reactor [38,39].

For greater clarity on how the four types of bioreactor configurations described above compare, Table 1 summarizes the working principles, advantages, and disadvantages of each.

### 2.2. Key Process Parameters

**Figure 4:**
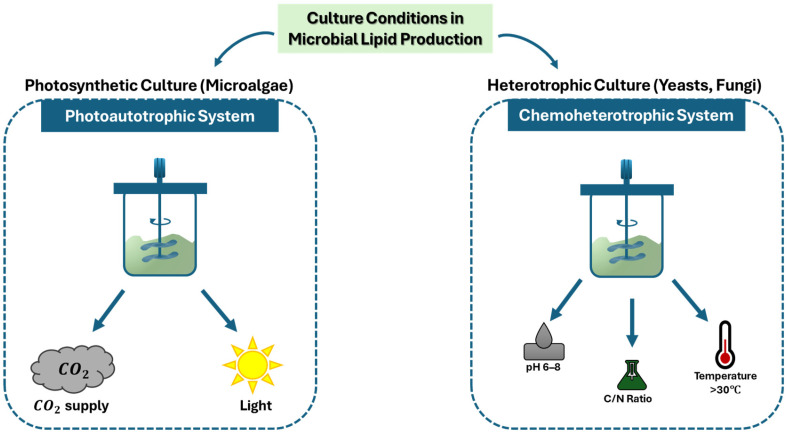
Schematic representation of the parameters implied in photosynthetic and heterotrophic culture systems for microbial lipid production.

The quality of microbial oils is influenced by the culture conditions of the microorganisms, including light intensity, temperature, pH, carbon dioxide (CO_2_) supply, culture medium composition, and growth cycle [1]. Figure 4 illustrates a classification of parameters for photosynthetic and heterotrophic culture systems used for microbial lipid production.

It has been demonstrated that light stress, an important abiotic factor, increases lipid production in several microorganisms, including microalgae. Multiple studies have shown that light modulation can enhance lipid synthesis in oil-producing bacteria [41]. Apart from color, light intensity is important for photosynthesis, which in turn affects microalgae growth and lipid accumulation. In numerous algal strains, an increase in light intensity can be positively associated with lipid content above a specific threshold, which differs among species [42]; for example, *Scenedesmus obliquus* showed a significant increase in lipid and protein levels at a light intensity of 150 µmol photons m^2^/s, demonstrating that light treatment is an effective method for enhancing lipid storage in these species [43].

pH is considered a fundamental parameter that regulates cellular metabolism and biomass generation in microalgae. The proliferation of microorganisms is significantly affected by pH, as it directly influences the NH_3_/NH_4_^+^ equilibrium and nitrogen availability. For optimal development, distinct microorganisms require specific pH ranges, and fluctuations in ambient pH affect their activity, especially in enzyme and protein activation. Although most microalgae favor neutral pH, certain species have been shown to flourish in highly acidic (pH < 5) or alkaline (pH > 9) conditions; for example, in a mixed microalgae culture, during the growth phase, an increase in biomass production and intracellular carbohydrate accumulation was observed. In contrast, significant lipid productivities (Total/neutral, 26.93/10.3%) were recorded during the starvation phase at pH 8 [44]. The growth of microalgae can alter the medium pH, thereby influencing microalgal development. An examination of proton imbalance during microalgae cultivation revealed that alkalinity was generated or consumed depending on nitrogen availability and metabolic processes, significantly affecting the medium’s pH more than CO_2_ absorption [45,46,47].

Temperature significantly influences microbial lipid production, serving as a crucial regulator of enzyme activity, growth rates, and membrane fluidity. Optimal temperature ranges differ among species, typically resulting in elevated saturated fat levels at higher temperatures for stability and increased unsaturated fat levels at lower temperatures to preserve fluidity. However, extreme heat can inhibit overall lipid synthesis [48,49]. For example, the optimal temperature for lipid production in *Streptomyces jeddahensis* was 37 °C, and sodium octanoate produced the highest lipid concentration. Significant lipid production continued at 45 °C despite growth declining, suggesting an adaptive stress response [50]. A decrease in temperature typically leads to a reduction in the average length of fatty acid chains, accompanied by an increase in methyl branching, unsaturated fatty acids, and the anteiso-to-iso fatty acid ratio. A contrasting trend has been observed in bacteria exposed to extremely low temperatures, marked by a greater ratio of saturated to unsaturated and branched fatty acids, which challenges the generalization of bacterial trends and suggests a species-specific response to temperature [51,52].

CO_2_ is crucial for regulating microalgal development by affecting the production of key components, which constitute approximately 50% of the biomass by weight. Elevated CO_2_ concentrations can promote microalgal proliferation, as the existing CO_2_ levels in saltwater are insufficient for optimal microalgal photosynthesis [53,54,55]. Microalgae generally increase lipid synthesis when exposed to enhanced CO_2_ concentrations; for instance, CO_2_ concentrations of 5% and 10% increased lipid content in *Skeletonema costatum* by 70% and 87%, respectively, compared with the ambient condition (0.04% CO_2_) [56]. Another study showed that the overall lipid content in microalgal–bacterial granular sludge (MBGS) with CO_2_ supplementation increased by approximately 46% than without CO_2_ supplementation. The phosphate removal efficiency in the experimental group with increased CO_2_ was considerably enhanced, enabling phosphate absorption by the microalgae in MBGS and subsequent conversion into lipids [57]. A comparable trend is noted in fatty acid composition; for example, the best conditions for maximizing lipid content and biomass concentration in the native green microalgae *Chlorella sorokiniana* were achieved with 20% CO_2_ [58]. In addition, in *Chlorella vulgaris*, elevating the CO_2_ concentration from 0.04% to 15% resulted in an augmentation of total fatty acid content. The stimulatory effects of increased CO_2_ on lipid and fatty acid production can be attributed to two factors: increased CO_2_, which can augment cellular carbon availability and is essential for lipid production in microalgae; and excessively elevated CO_2_ concentrations, which can induce growth arrest and promote fat accumulation in algal cells [59,60].

The operational and environmental parameters during biomass production, along with the culture medium composition, considerably influence biomass efficacy and the synthesis and accumulation of metabolites. The composition of the culture medium is crucial, as it can alter biomass composition and prevent the proliferation of undesirable contaminants. The primary constituents of microalgal biomass include oxygen (30–50% dry weight—d.w.), carbon (30–50% d.w.), nitrogen (4–9% d.w.), hydrogen (3–7% d.w.), and phosphorus (1–3% d.w.), along with trace amounts of elements such as sulfur, magnesium, potassium, and calcium [61,62]. Nitrogen deprivation is an efficient method for enhancing lipid production in microalgal cells. Nitrogen-deficient culture conditions yielded twice the lipid production as nitrogen-sufficient medium [63]. Zarrinmehr et al. [64] reported that high and low nitrogen concentrations in the growth medium induced stress in microalgae cells. Moreover, higher concentrations of nitrogen (from 72 mg/L to 144 mg/L) could increase biomass production in *Isochrysis galbana*. This finding aligns with that of Feng et al. [65], who observed that in nitrogen-deficient environments, *Chlorella zofingiensis* could produce 65% lipids. Phosphorus represents an essential element for the growth and cellular division of microalgae, with its importance varying among different species. Roopnarain et al. [66] indicate that the ideal phosphorus concentration for maximum biomass productivity lies between 0.001 g/L and 0.179 g/L. Phosphorus limitation is a significant environmental constraint on lipid formation. *Scenedesmus* sp. boosted cellular lipid level, which subsequently reduced total lipid production during phosphorus deprivation. *Scenedesmus* sp. cultivated in 50 mg/L phosphorus attained a lipid content of 22.3%, while lipid yield achieved 42.5% in 1 mg/L phosphorus [67]. Thus, nitrogen starvation and phosphate repletion represent the most effective strategies for lipid accumulation. A maximum lipid productivity of 82.0 mg L^−1^ day^−1^ was achieved for *Chlorella vulgari* under N−P+ conditions, which was 1.5 times and 4.6 times higher than the amounts produced when both nutrients were limited and when both N and P were replete, respectively [68].

The growth cycle significantly influences microbial lipid production, with distinct phases promoting either biomass growth or lipid accumulation, often leading to a transition from polar lipids to neutral lipids, thereby optimizing oil content as cultures mature and nutrients diminish. A two-stage strategy, in which cells are initially cultivated, and lipid synthesis is induced by altered conditions (such as nutrient limitation or the addition of specific substrates), is a prevalent approach to enhance lipid yields for industrial applications such as biofuels and nutraceuticals, separating cellular growth from lipid accumulation [69].

Microbial lipids are typically synthesized under the conditions of sufficient C and limiting nutrients (N, P, or S), although biomass growth predominates when C and N are available [70]. A higher C/N ratio will facilitate lipid accumulation, whereas a lower C/N ratio will promote cell development. The type of nitrogen source also affects lipid production. Some yeast strains preferentially utilize organic nitrogen sources, such as yeast extract or peptone, while others perform better with inorganic sources, such as ammonium salts [71]. The metabolic process can be adjusted for lipid synthesis using biochemical engineering methods. Oleaginous microorganisms tend to turn surplus C into lipids when N is limited; for example, *Cunninghamella echinulata*’s lipid content might rise from 36% to 47% with an increase in the C/N ratio from 83.5 to 133.5 [1]. An increased C/N ratio, typically above 100, is essential for optimizing lipid synthesis. Nitrogen limitation, combined with an excess of carbon, facilitates the formation of TAGs as energy reserves [72]. When cultivated on xylose with a C/N ratio of 120, *Rhodosporidium toruloides* showed the ability to retain up to 60% intracellular lipids [73]. *Mucor irregularis* JR 1.1 produces more biomass and lipids when the C/N ratio fluctuates significantly. Maximum lipid production and biomass can be achieved with a C/N ratio of 40 and a 72 h incubation [74]. *Nannochloropsis oceanica* and *Tisochrysis lutea* are two potential microalgal strains that serve as environmentally friendly sources of n-3 long-chain PUFAs under mild nitrogen limitation. N-limitation led to TAG accumulation; however, the increased lipid content in microalgal cells during extreme N-limitation may not improve lipid efficacy, as biomass production is often reduced. Thus, a comprehensive approach incorporating metabolic engineering, selection, and training might prove essential to increase both lipid and n-3 LC-PUFA content without considerable biomass loss [75].

The selection of carbon sources significantly influences lipid production. Various carbon sources affect microbial metabolic pathways and lipid synthesis rates. Monosaccharides, such as glucose, and disaccharides, like sucrose, are metabolized more readily than polysaccharides, including starch. The carbon source strongly influences lipid production, with glycerol yielding more lipids than carbohydrates such as xylose and glucose. Glycerol can produce up to 69% of the absorbed carbon as lipids, rendering it an exceptionally efficient substrate for the synthesis of lipids [76]. The nitrogen source substantially impacts microbial lipid synthesis by altering growth mechanisms and lipid accumulation. Inorganic nitrogen sources, such as sodium nitrate (NaNO_3_), are more effective at enhancing lipid formation than organic sources such as urea or ammonium sulfate. *Crypthecodinium cohnii* demonstrated a lipid amount of 26.9% when cultured with NaNO_3_ [77]. It has been demonstrated that nitrogen feeding significantly affects *Yarrowia lipolytica* cell growth and microbial lipid synthesis during high-concentration acid cultivation. Under optimal culture conditions, the maximum microbial lipid production reached 4.00 g/L, with a dry cell weight of 9.91 g/L and a lipid content of 40.37%, predominantly C16-C18 fatty acids [78]. Traditional nitrogen sources such as ammonium sulfate and ammonium nitrate enhance lipid yields; however, urea has emerged as a sustainable alternative that yields similar results. For improving microbial lipid synthesis, urea, ammonium nitrate, sodium nitrate, and ammonium sulfate are considered optimal [79].

Because it participates in oxidative reactivity and enzymatic conversions, oxygen is essential for lipid metabolism. It is particularly important when genetic diseases impair cellular repair processes. Oxygen tension can trigger a fast adaptive response, shaped by physical conditions and the reconfiguration of the lipid pool, propelled by chemical (oxidative–redox), enzymatic, and metabolic processes that alter the availability of fatty acids for membrane phospholipid remodeling. Desaturase enzymes are essential in fatty acid synthesis and require an oxygen molecule, thereby enabling oxygen tension to influence the levels of unsaturated fatty acids (UFA). Investigating membrane lipidomics in cell culture under diverse conditions yields insights into the preservation and turnover of the cell membrane, an essential compartment for physiological function [80,81]. Although more than 90% of the oxygen utilized by living organisms is employed in energy production via oxidative phosphorylation via the electron transport chain, which employs a four-electron mechanism resulting in ATP and water synthesis, there remains a persistent risk of its transformation into reactive oxygen species (ROS) [82]. While excessive ROS can harm cellular components through oxidation, moderate levels of ROS are essential for efficient lipid formation. Menon et al. [83] discovered that the concentrations of cells and certain intracellular neutral lipid levels in *Chlorella vulgaris* are inversely and directly associated with specific intracellular ROS levels, following a power law connection. Furthermore, a suitably calibrated exposure to exogenous ROS, such as hydrogen peroxide (H_2_O_2_), that facilitates the generation of such ROS might induce the synthesis of neutral lipids. *Scenedesmus* sp. subjected to oxidative stress exhibits an increased formation of total lipids, with minimal variation observed after 24 h of H_2_O_2_ treatment. Despite the inhibition of algal development from the application of various treatments, the proportion of C16:0 fatty acid content grew, whereas that of C18:3 fatty acid declined simultaneously [84].

The variation in the reported C/N ratio (40–120) for microbial lipid production is mainly attributed to differences in microbial physiology, substrate characteristics, and cultivation strategies. Oleaginous microorganisms exhibit distinct metabolic responses to nitrogen limitation, and the threshold at which carbon flux is redirected toward lipid biosynthesis varies between species due to differences in enzymatic activity and regulatory mechanisms. In addition, substrate type significantly affects the effective C/N ratio, as carbon sources such as glucose, glycerol, or lignocellulosic hydrolysates differ in uptake rates and metabolic efficiency, thereby influencing lipid accumulation [13,85,86]. Fermentation also plays a key role; in batch systems, nitrogen depletion occurs rapidly, whereas in fed-batch systems, the controlled addition of nutrients alters the dynamic C/N balance, leading to different optimal ranges [26]. Therefore, the wide variation in optimal C/N ratios reported in the literature reflects the combined effects of strain specificity, substrate variability, and process conditions rather than inconsistencies in experimental data.

## 3. Adaptive Laboratory Evolution in Support of Bioprocess Robustness

Adaptive laboratory evolution (ALE) is a non-genetically modified organism (non-GMO) method for enhancing microbial strains. ALE is an important technique that enables the selection of strains with enhanced characteristics from a developing population by artificially replicating the mutation and selection processes found in natural environments in laboratory settings [87,88]. This method has been used to improve fitness under atypical conditions, such as immunity to toxic solvents and other byproducts that impede industrial fermentation, as well as increased temperatures, which may be advantageous for minimizing downstream separation costs or reducing contamination risks [89]. ALE promotes tolerance to hypoxia or elevated carbon-to-nitrogen (C/N) ratios by simulating natural selection in a laboratory setting, compelling microbes to attain advantageous mutations through iterative cycles of proliferation and selection [87,90]. For example, ALE significantly improved essential attributes of *Lactobacillus casei*, including enhanced antioxidative capabilities and other associated stress-resistant probiotic qualities. These changes establish *L. casei* as a viable probiotic for lowering oxidative stress, while circumventing issues related to genetic alteration. The modified strain possesses possibilities as an active antioxidant, capable of mitigating oxidation and inhibiting the accumulation of harmful chemicals in fermented foods [91]. Moreover, ALE is a technique for rapidly generating advantageous phenotypes through metabolic and transcriptional reconfiguration. Hirasawa et al. [92] reported the implementation of ALE for the effective fermentation of the *Saccharomyces cerevisiae* strain CEN.PK113-7D, utilizing an imbalanced C/N medium to exert selective pressure. The group identified multiple evolved strains that exhibit a 3–5-fold increase in amino acid utilization and ammonia excretion. The multi-step bioreactor approach utilizing the developed strain AAV6, enhanced with concentrated nitrogen supplies, yielded 1.36 g/L of ammonia, consistent with outputs from previous microbial systems.

Although it is effective, ALE encounters significant technical constraints that limit its wider industrial use. ALE generally necessitates prolonged evolutionary cycles, as fitness improvements tend to slow over time due to diminishing returns and escalating network complexity, rendering the method both time- and resource-intensive. The genetic stability of evolved phenotypes is a significant concern, as ALE frequently selects context-dependent mutations that may lack robustness under fluctuating environmental conditions or during scale-up. Additionally, population bottlenecks and selection bias favoring dominant lineages can result in the loss of advantageous low-frequency variants and diminished reproducibility. Third, identifying causal mutations is inherently difficult, as ALE usually produces numerous mutations across both coding and non-coding regions, with intricate interactions among them [93].

Conversely, GMO-driven synthetic biology methodologies, particularly the latest advancements in CRISPR/Cas9 technology, provide effective and scalable genome editing, encompassing multiplexed adjustments. These advancements are anticipated to accelerate product development, reduce costs, and improve environmental sustainability. The primary applications in bacteria, yeasts, and filamentous fungi are represented by gene elimination, integration, and expression regulation. In collaboration with process engineering, CRISPR/Cas9 technology enhances metabolic pathway optimization, increases resilience to fermentation stressors, and facilitates dynamic control of bioprocesses to enhance productivity [91,94,95]. CRISPR-edited strains have demonstrated enhanced stability and productivity throughout extended fermentation processes by improving tolerance to pH changes, osmotic stress, and elevated product concentrations. These characteristics are especially crucial for fed-batch and batch fermentation systems typically employed in industrial environments. CRISPR-enabled regulatory circuits facilitate the dynamic modulation of metabolic activity in response to environmental variables. These advancements facilitate the creation of smart fermentation systems that can sustain optimal performance under diverse operational conditions [96]. Integrating ALE with high-throughput genome sequencing, CRISPR-mediated reconstruction, and automated culture systems can help to overcome these bottlenecks by accelerating evolution, stabilizing beneficial traits, and pinpointing key genetic targets.

### 3.1. Stressors Used in ALE

ALE employs diverse stressors to select microbes exhibiting improved characteristics, typically involving toxic substances (ethanol, acetic acid, furfural, H_2_O_2_), nutrient low availability, extreme pH levels, increased salinity, thermal stress (heat, freeze–thaw cycles), oxidative stress, and specific carbon monoxide concentrations, compelling microbes to adapt for enhanced survival and efficacy in demanding industrial or natural settings [87,97,98]. For an at-a-glance perspective, Table 2 summarizes the stressors used in ALE and the resulting beneficial phenotypes.

To induce thermal stress, researchers incrementally raise incubation temperatures (mesophilic organisms have T_OPT_ between 25 and 45 °C), compelling microbes to develop enhanced thermal tolerance, resulting in strains that are better suited for industrial applications such as biofuel production or optimized fermentation [99,100]. The application of a ratcheting ALE procedure elevated the temperature tolerance of *Nitzschia inconspicua* from 35.5 °C to 37.5 °C after more than 300 days of flask growing. This adaptation indicates a systematic increase in the upper temperature limit for this organism, with transformed lines able to withstand temperatures lethal to the naturally occurring wild-type strain. Numerous high-temperature clonal isolates were obtained from the developed population during ALE, and increased temperature tolerance was observed in the clonal, non-clonal, and parental-adapted cultures. The ALE method enables the enhancement of outstanding industrial algae strains without producing genetically modified organisms (GMOs) [101]. Rychel et al. [102] employed ALE to engineer *Escherichia coli* strains that survive at 45.3 °C, a temperature lethal to wild-type cells. The strains had a hypermutator phenotype and employed numerous system-level alterations, hindering the comprehensive analysis of DNA mutations. The group was required to clarify the adaptive mechanisms for extreme temperature tolerance at the transcriptomic level. They used independent modulated gene set (iModulon) analysis to identify five transcriptional pathways that promote growth at elevated temperatures. These pathways were linked to acquired mutations, changes in transcriptome composition, sensory information, phenotypes, and protein structure. Li et al. [103] obtained *Corynebacterium glutamicum* strains with increased tolerance to elevated temperatures. The genomic analysis indicated that the advanced strains possessed 13 missense mutations and 3 synonymous mutations compared to the non-evolved parent strain. Additionally, reverse transcription quantitative PCR (RT-qPCR) study of the hrcA-L119P mutant demonstrated that both groEL genes were upregulated at 42 °C, promoting the emergence of robust strains with improved heat resistance. Furthermore, a significant increase in FAS-IA and FAS-IB transcription in the fasR-L102F strain is essential for protecting cells from thermal shock.

**Table 2:** Stressors used in ALE and the resulting beneficial phenotypes.

Stressor	Target Organism	Effect on Lipid Accumulation and Relevant SCOs	Improved Phenotype	Industrial Benefit	Ref.
High temperature	Nitzschia *inconspicua*	Increased lipid accumulation stability under heat stress; maintenance of TAG productivity	Enhanced thermal tolerance; Stable heat tolerance across isolates; Improved robustness under heat stress	Improved outdoor cultivation viability; Reduced culture crashes; Lower operational costs	[101]
	*Escherichia coli*	Not directly relevant (non-oleaginous model; potential platform for engineered lipid pathways)	Extreme thermotolerance	Reduced contamination risk; Potential for new strain engineering targets	[102]
	*Corynebacterium glutamicum*	Potential improvement in lipid-derived metabolites; increase in fatty acids content	Enhanced thermotolerance; Improved cellular protection; Genetically stable adaptive changes	Faster and more efficient high-temperature fermentation; Reduced cooling and energy costs; Increased process robustness	[103]
Oxidative stress	*Lactococcus lactis* subspecies *lactis* LM1009	Enhanced cyclopropane fatty acid (CFA) synthesis after oxidative adaptation	Cross-protection against multiple stresses; Improved functional and probiotic traits; Reprogrammed redox metabolism; Anti-inflammatory bioactivity	Next-generation probiotic development; Improved manufacturing robustness; Health-oriented functional foods and supplements	[104]
	*Lacticaseibacillus rhamnosus*	Not relevant for SCO (probiotic, non-lipid accumulating organism)	Enhanced oxidative stress tolerance; Faster adaptation and growth under oxidative stress	More robust probiotic strains; Cost-effective and scalable strain development; Improved functional food performance	[105]
Fermentation byproducts	*Saccharomyces cerevisiae*	Indirect: increased storage lipids (TAGs) linked to stress tolerance and carbon storage	Enhanced fermentation stress tolerance; Increased intracellular carbohydrate storage; Improved fermentative performance	Higher ethanol yields and productivity; More robust and reliable fermentations; Improved process economics	[106]
Inhibitors	*Escherichia coli*	Not native SCO producer; potential chassis for ester/lipid pathway engineering	Enhanced isobutyl acetate (IBA) tolerance; Metabolic and regulatory optimization	Elimination of complex phase-separation strategies; Higher product titers; Lower production costs and improved scalability	[107]
	*Paecilomyces variotii*	Limited SCO relevance; possible minor lipid metabolism involvement during detoxification	Enhanced tolerance and assimilation of lignocellulose-derived inhibitors; Improved biodetoxification capacity; Upregulated detoxification and assimilation pathways	Effective biodetoxification of lignocellulosic hydrolysates; Higher downstream product yields; Improved biorefinery efficiency	[108]
	*Saccharomyces cerevisiae*	Indirect: improved redox balance may support lipid biosynthesis under inhibitor stress	Enhanced furfural tolerance; Shortened lag phase; Increased ethanol production; Upregulated detoxification genes	Higher ethanol yield from biomass; Shorter fermentation time; Cost-effectiveness	[109]
	*Rhodosporidium toruloides*	Increased lipid accumulation under inhibitor stress; improved SCO yield from lignocellulosic substrates	Enhanced tolerance to biomass-derived inhibitors; Improved growth under inhibitory conditions	More efficient biomass hydrolysate fermentation; Potential for stable large-scale bioprocesses	[110]

Abbreviations: SCO—Single-Cell Oil; TAG—triacylglycerol.

Oxidative stress exerts a significant selective pressure in ALE, favoring microbes with enhanced resilience, simulating industrial or extreme environmental conditions to enhance traits for biotechnology, probiotics, or food safety, often by promoting antioxidant enzyme activity, stress-response proteins, and metabolic alterations that improve survival and functionality [111]. *Lactococcus lactis* subsp. *Lactis* LM1009 was subjected to progressively elevated hydrogen peroxide concentrations over 50 days, yielding an adapted strain capable of enduring up to 5 mM hydrogen peroxide. Compared to the original strain, *Lactococcus lactis* subsp. lactis LM1009-A (adapted strain) exhibited enhanced resistance to oxidative, acidic, and thermal stress, along with heightened surface hydrophobic properties, auto-aggregation characteristics, and intestinal adhesion capacity. This highlights its potential as a future probiotic for the management of oxidative and inflammatory stress conditions, in addition to industrial applications. The antioxidant activity was significantly improved, as demonstrated by higher scavenging of 2,2-diphenyl-1-picrylhydrazyl radicals, enhanced inhibition of linoleic acid peroxidation, and reduced levels of intracellular reactive oxygen species (ROS) [104]. Microfluidic platforms complement large-batch ALE cultures, offering enhanced cost-effectiveness and efficiency. Microfluidic ALE platforms exhibit potential; however, many systems have suffered from inadequate cell passaging and suboptimal spatial resolution. A novel methodology is introduced utilizing a microfluidic Evolution on a Chip (EVoc) design that systematically transports microbial cells from regions of lower H_2_O_2_ concentration to regions of higher concentration. Extended exposure, lasting up to 72 h, demonstrated the viability of adaptable strains of *Lacticaseibacillus rhamnosus*, a helpful probiotic frequently used in food products. The adaptive strains exhibit distinct morphology and gene expression patterns relative to the wild-type, and genome sequencing has identified a potentially significant single-nucleotide mutation in the omega-amidase gene [105].

ALE is frequently employed to simulate organisms, including *Saccharomyces cerevisiae*, *Bacillus subtilis*, and *E. coli*, due to their manipulability and established fermentation techniques [87,92,112]. Batch cultivations of wild and modified *S. cerevisiae* were conducted to assess the process of fermentation kinetics and carbohydrate storage in an associated manner. The adapted strain’s maximum trehalose and glycogen levels (7.5% and 10.5%) were noticeably higher than those of the wild strain (5% and 6.5%). The modified *S. cerevisiae* exhibited improved resistance to fermentation stressors, leading to increased ethanol productivity (2.03 vs. 1.65 g ethanol/h) and an elevated ethanol titer (7.7 vs. 6.4%). This investigation enhances understanding of the cellular response of *S. cerevisiae* to fermentation challenges related to culture history, alongside the assessment of fermentation performance and process kinetics [106]. ALE was utilized on *E. coli* to enhance isobutyl acetate (IBA) tolerance, thereby circumventing an expensive and intricate approach. After 37 rounds of selection pressure, 22 IBA-tolerant mutants were identified. The resulting mutants not only withstand elevated IBA concentrations but also generate increased IBA titers. Whole-genome sequencing, coupled with CRISPR/Cas9 genome editing, identified the mutations that enhance tolerance and increase titers. The enhanced IBA titers in the evolved mutants resulted from an augmented supply of acetyl-CoA and modified transcriptional machinery. By combining specific advantageous mutations, a strain that could produce 3.2 times more IBA than the parent strain was created without the need for phase separation [107].

ALE for non-GMO strains uses natural selection in laboratory settings to cultivate microbes with advantageous traits (such as enhanced stress tolerance or yield) without direct genetic alteration, making them suitable for industrial applications. Patzschke et al. [113] employed acrolein as a selection agent to isolate strains that overproduce glutathione via ALE under progressively increasing doses. Nevertheless, because factors beyond glutathione also influence acrolein resistance, the ALE method often yielded strains with reduced glutathione levels. To overcome this constraint, a mutant library was created via UV radiation and identified mutants exhibiting elevated glutathione levels through an acrolein resistance-mediated screening method [114].

### 3.2. Phenotypes That Benefit Industrial Processes

Recent studies have shown the effectiveness of the ALE method in modifying algal metabolite composition under various stress circumstances. For example, *Phaeodactylum tricornutum* cells exposed to dual-factor ALE showed a strong preference for food waste hydrolysate as a carbon source, leading to enhanced biomass and PUFA production. ALE, under high-temperature conditions, enhanced cellular growth and lipid synthesis while mitigating the effects of oxidative stress in Schizochytrium sp. *Chlorella vulgaris* has attracted significant interest among microalgae due to its notable biological traits, including a rapid growth rate, oleaginicity, high photosynthetic efficiency, and the ability to assimilate nutrients from wastewater. Consequently, there is an urgent need to develop rapidly proliferating algae species, such as *C. vulgaris*, which may yield strains with enhanced lipid productivity. However, limited reports exist despite their biotechnological promise [115,116,117].

The expanded utilization of feedstock in ALE emphasizes the development of microbes capable of efficiently processing complex, low-cost, or waste materials rather than simple sugars. This approach facilitates sustainable bioproduction of fuels, chemicals, and proteins by improving tolerance to inhibitors and uncovering new metabolic pathways, often employing automation and genome sequencing to expedite strain optimization [118]. Increased inhibitor concentrations in lignocellulosic feedstock adversely impact the breakdown rate of biodetoxification strains. Two versatile laboratory evolutions in solid substrate and liquid medium were conducted to enhance the biodetoxification potential of *Paecilomyces variotii* against elevated concentrations of lignocellulose-derived inhibitors, yielding two evolved strains, AC70 and ZW70. The findings indicated that evolutionary change in liquid medium enhances acetic acid assimilation more effectively than in a solid substrate. The ZW70 strain can efficiently eliminate the high concentration of inhibitor cocktails from hydrolysates obtained from pretreated wheat straw and furfural residues. The biodetoxified hydrolysates produced by ZW70 were effectively used to manufacture chiral L-lactic acid from cellulose, achieving titers of approximately 110 g/L, which exceeded those obtained with the parental strain by over 20% [109].

Improving microbes’ resilience to inhibitors is an essential strategy. In contrast to traditional technologies, ALE is not constrained by the physiological processes behind microbial phenotypes for enhancement. Furfural exhibits inhibitory effects on *S. cerevisiae*. To investigate the mechanism underlying augmented furfural tolerance, the ADR1_1802 mutant was engineered using CRISPR/Cas9, supported by whole-genome resequencing data. The results demonstrated that the onset of growth for ADR1_1802 was expedited by 20 h relative to the reference strain (*S. cerevisiae* CEN.PK113-5D) at a furfural concentration of 4 g/L. This finding indicates that the mutant’s increased furfural tolerance results from accelerated furfural breakdown [109]. In another study, Liu et al. [110] examined the effects of various biomass-derived inhibitors on *Rhodosporon* proliferation; specifically, furfural, 5-hydroxymethylfurfural (5-HMF), acetic acid, vanillin, and vanillic acid. The results indicated that furfural had the most potent inhibitory effect, followed by vanillin and 5-HMF.

### 3.3. Examples and Case Studies

Oleaginous yeasts, including *Rhodosporidium toruloides*, *Yarrowia lipolytica*, *Rhodotorula glutinis*, and *Lipomyces starkeyi*, constitute a new frontier in renewable energy research, highlighting the crucial significance of microbial lipids in the future of sustainable biofuel development [119].

*Y. lipolytica* represents a non-pathogenic yeast classified as GRAS (Generally Recognized as Safe) by the FDA. This yeast can accumulate lipids, comprising 40% of its total dry biomass composition. In nutrient-deficient conditions, fat accumulation may exceed 70% of biomass. Y. lipolytica efficiently metabolizes hydrophobic substrates, making it beneficial to produce SCO, single-cell protein (SCP), and citric acid, which has fostered interest in research focused on supplying raw materials for biofuel generation and various industrial applications [120,121,122].

The oleaginous yeast *Y. lipolytica* serves as a promising platform for lipid biosynthesis and utilization in food technology. Nevertheless, its lipid accumulation has historically depended on nitrogen limitation, making it difficult to attain high yields in nutrient-rich environments. Lee et al. [123] modified *Y. lipolytica* to improve productivity and fat accumulation in nutrient-rich conditions. Substantial modifications included the removal of MHY1 to suppress filamentous development, the overexpression of TAG biosynthetic genes, a breakdown of fatty acid degradation, and the rerouting of phosphatidic acid flow towards TAG biosynthesis by reducing phospholipid production through OPI3 deletion and CDS1 mutation. Furthermore, the deletion of CEX1 to suppress citrate excretion significantly enhanced lipid accumulation. The strain CJ0415 yielded 54.6 g/L of lipids, exhibiting a lipid content of 45.8% and a lipid efficiency of 2.06 g/L/h under nutrient-rich conditions in a 5 L fermenter, reflecting a 2.6-fold increase compared to nitrogen-limited conditions. These findings underscore the potential of Y. lipolytica as a solid foundation for large-scale industrial lipid production in nutrient-rich conditions.

Fabiszewska et al. [124] aimed to characterize biomass to assess its nutritional properties and suitability for food applications. In addition to significant protein content (24.16–27.03%) and unsaturated fatty acids, such as oleic acid (62.73–66.44%) and linoleic acid (19.40–21.40%), the biomass of *Y. lipolytica* showed a significant lipid content (39.43–50.53%). Lipid-rich biomass produced in cultures with shorter time frames (20 h), concluding in the logarithmic growth phase, exhibited lower oxidative stability in comparison to the longer cultures (65 h) that finished in the stationary growing phase. The biomass of *Y. lipolytica*, which comprises roughly 20% lipids, may serve as a feasible raw material for food science and technology, providing an environmentally friendly substitute for traditional vegetable oils.

Kurt et al. [125] demonstrated the potential of integrating model-guided continuous fermentation using adaptive learning environments in a continuous-flow bioreactor to enhance lipid synthesis by the modified yeast *Y. lipolytica* E26. Fed-batch fermentation identified appropriate pH ranges for enhanced lipid production and provided essential insights into the strain and fermentation method, thereby aiding the design and optimization of continuous fermentation (CF) for improved lipid productivity. A kinetic model simulation was utilized under diverse feed and operational conditions to determine the earliest process parameters used for the CF in the 1 L bioreactor investigation. Compared to the fed-batch fermentation method, the single-stage CF significantly increased lipid productivity from 0.3 to 0.5 g/L/h to around 1 g/L/h, while maintaining lipid yield at around 0.1 g/g. Moreover, the long-term CF-produced mutants displayed significantly faster cell proliferation, improved lipid synthesis, and diminished byproduct generation, illustrating the efficacy of utilizing ALE in a continuous bioreactor to extract mutant strains with considerably enhanced lipid production.

*Rhodosporidium toruloides*, a red yeast, by nature synthesizes carotenoids and microbial lipids. Over the past decade, numerous investigations have established *R. toruloides* as a potential platform for lipid synthesis due to its varied substrate affinities, strong stress resistance, and other advantageous characteristics [126]. Wankhede et al. [127] investigated the utilization of wood hydrolysate, a widespread and economical lignocellulosic substrate, as the principal source of carbon for lipid and carotenoid synthesis by *R. toruloides*-7191 in fed-batch cultivation within a 3 L bioreactor. The fed-batch strategy, selected over batch and continuous methods, facilitates regulated nutrient provision, reduces substrate inhibition, and preserves an optimal C/N ratio, thus promoting extended biosynthesis and enhanced product yields. The procedure attained a peak lipid output of 22.33 g/L, a cumulative lipid accumulation of 57.9%, and a total carotenoid yield of 4.23 mg/L. Fatty acid analysis reveals a composition abundant in oleic acid (C18:1), linoleic acid (C18:2), and palmitic acid (C16:0), signifying its appropriateness for biodiesel applications.

Another study used sustainable tea waste hydrolysate (TWH) as the sole carbon source for the production of carotenoids and lipids. A novel mutant strain of *R. toruloides*, designated RM18, was identified via atmospheric and ambient temperature-controlled plasma mutagenesis, followed by continuous domestication in tea waste hydrolysate derived from *R. toruloides* ACCC20341. RM18 produced greater biomass and higher levels of carotenoids and α-linolenic acid than the control strain cultivated in tea waste hydrolysate. Furthermore, α-linolenic acid synthesis from RM18 in TWH constituted 5.5% of total lipids, which was 1.58 times greater in comparison to that of the control strain. The *R. toruloides* mutant RM18 demonstrated significant carotenoid and lipid production when cultivated in tea waste hydrolysate, rendering it suitable for industrial uses [128].

The oleaginous yeast *Lipomyces starkeyi* constitutes a promising lipid producer capable of accumulating up to 70% of SCO relative to its dry cell weight under optimal conditions. Compared with other oleaginous organisms, it can be cultivated on a broad range of feedstocks, and significant quantities of the generated lipids can be converted into biodiesel [129]. Martinez-Burgos et al. [130] evaluated soybean hulls as sources of carbon for biolipid synthesis by *L. starkeyi* LPB 53. The hulls were initially subjected to physicochemical and enzymatic treatment to extract fermentable carbohydrates. Thereafter, biomass proliferation was assessed using various nitrogen sources, and lipid production was optimized, achieving a peak cell biomass concentration of 26.5 g/L and 42.5% lipid content. Approximately 65% of the xylose content was used. The extracted oil predominantly comprised oleic, palmitic, palmitoleic, linoleic, and stearic fatty acids in proportions of 54%, 32%, 4%, 3%, and 2%, respectively.

Rachmadona et al. [131] assessed the efficacy of the oleaginous yeast *L. starkeyi* strain D35 in lipid synthesis when grown on palm oil mill effluent (POME), a widely utilized and underexploited waste product. As a novel carbon source, it thereby illustrates its capacity for substantial lipid accumulation and presents an innovative method for waste valorization and sustainable bioprocessing. *L. starkeyi* D35 was cultivated under three conditions—glucose alone, POME alone (5 g/L), and a combination of glucose and POME—then fermented at 190 rpm and 30 °C for 96 h. The strain attained substantial lipid production and vigorous growth, achieving a lipid content of 31.81 g/L. Analysis indicated that the predominant intracellular lipids were C16 and C18 fatty acids, suggesting their suitability for biofuel applications.

While yeasts primarily manufacture oleic acid (OA, 18:1 n9), filamentous fungi of the *Mortierellaceae* family are known for their PUFA profile, especially their ability to synthesize long-chain fatty acids like arachidonic acid (ARA). ARA, dihomo-gamma-linolenic acid (DHGLA, C20:3, n6), linoleic acid, gamma-linolenic acid (GLA, C18:3, n6), and eicosapentaenoic acid are among the fatty acids produced by fungi belonging to the *Mortierellaceae* family. These chemical acids promote susceptibility to phytopathogens in plants and are commonly used in dietary supplements and medications to augment immune responses in humans [132,133].

Chen et al. [134] illustrated that *Mortierella alpina* FU30797 is considered suitable for the synthesis of PUFA-ARA and established that taro processing waste hydrolysate can function as an effective low-cost substrate for cultivating *M. alpina* FU30797. They proposed the utilization of a sea salt solution as an alternative to seawater, with the aim of conserving freshwater resources. This study’s findings may provide relevant insights into enhancing key metabolites in the PUFA-ARA biological process, highlighting sustainability and biorefinery.

ARA is an essential constituent of cellular structures and is essential for the functioning of the neurological, muscle, and immunological systems. The urgent need for this nutrient may be met by cultivating the fungus *M. alpina*. Liquid media containing glucose and peptone as carbon and nitrogen sources, subsequently, along with several micronutritional variables, were optimized for maximal biomass and ARA production. Shaking flask cultivation at 25 °C for 7 days yielded approximately 0.570 g of ARA per liter of culture. The treatment of *M. alpina* with the mutagen 5-fluorouracil and octyl gallate-supplemented glucose–yeast–agar screening plates, followed by shake-flask incubation at 25 °C, then at 20 °C, and aging at 10 °C, resulted in over 3 g of arachidonic acid per liter of culture, a yield evaluated as appropriate for prospective commercial production [135].

Moreover, treatment with 5-fluorouracil (100 µg/mL) alone was the most effective in producing an isolate with increased lipid, PUFA, and ARA contents compared with diethyl sulfate and the combination of octyl gallate and nocodazole. The isolate M5F047 and the parent strain *M. alpina* demonstrated arachidonic acid and PUFA contents of 12.9% and 26.7%, and 13.6% and 7.7%, respectively. These values nearly doubled the PUFA content of the parent strain and exceeded the arachidonic acid content by 1.5 times in a 1 L fermentation conducted over 3 days. This was accomplished with the eighth regeneration of the isolate, demonstrating its durability and potential as an addition to plant-based meats to enhance meaty flavor, customer acceptance, and the overall experience. *M. alpina* ARA oil has attained Generally Recognized as Safe (GRAS) status, and the isolation enhancements through random mutagenesis applied herein circumvent the commercialization challenges linked to genetically modified organisms [136].

## 4. Substrate Engineering and Waste Valorization

Using agro-industrial food residues to develop industrially important enzymes offers a cost-effective route for producing value-added chemicals. Recent advances focus on improving catalytic efficiency or creating novel enzymes with unique characteristics. Challenges such as low activity and enzyme denaturation can be mitigated using affordable immobilization carriers, including agro-food materials, biopolymers, and nanomaterials [137,138]. To reduce production costs, renewable, low-cost carbon sources are explored for lipid production by oleaginous microorganisms [1,138,139].

Glucose is the primary substrate used by oleaginous microorganisms for lipid production, as it is the most appropriate carbon source for microbial growth. Nonetheless, the cost of large-scale lipid manufacturing using glucose as a feedstock is substantial [70,140].

The use of lignocellulosic feedstocks is a promising, sustainable, and environmentally friendly approach. Nonetheless, lignocellulosic biomass requires acidic thermochemical pre-treatment to obtain enhanced sugar yields for microbial utilization [141]. This step enhances the accessibility of cellulose to enzymatic hydrolysis [142]. The intensity of acid pretreatment, characterized by higher temperatures or higher acid concentrations, is positively associated with the production of fermentation inhibitors. Furan analogs and phenolic compounds are significant inhibitors of microbial fermentation. Pre-treatment of lignocellulosic biomass using chemical, physical, or biological methods enhances cellulose accessibility. Acid pre-treatment effectively eliminates hemicellulose and lignin while enhancing cellulose hydrolysis; however, it generates fermentation inhibitors that reduce fermentation efficiency and complicate subsequent processing. Pre-treatment of lignocellulosic feedstock alters its structure and leads to the formation of various inhibitors, including furans formed from the excessive decomposition of glucose and xylose, weak acids produced during hemicellulose degradation, and fatty acids resulting from the transformation of furfural [143,144].

Chitin consists of N-acetyl-D-glucosamine (GlcNAc) units connected by β1,4-glycosidic bonds. It is the second most prevalent biomass in the biosphere, after lignocellulose. Chitin is widely found in prawn shells, insect exoskeletons, fungal cell walls, and squid, with an annual production estimated at approximately 6–8 million tons [145]. Oleaginous species have been found to have limited ability to assimilate chitin directly for lipid production, attributed to the absence of relevant hydrolase genes and metabolic capacity. GlcNAc, the fundamental structural unit of chitin, can be utilized for lipid biosynthesis by certain oleaginous yeasts, including *Cutaneotrichosporon oleaginosum* (formerly *Cryptococcus curvatus*), *Trichosporon fementans*, *Trichosporon cutaneum*, and *Cryptococcus albidus*. The effective use of chitin materials necessitates their initial degradation. Different depolymerization strategies will release different fermentable carbon sources from chitin. The enzymatic degradation of chitin has been extensively studied to produce GlcNAc. This method is still in the laboratory phase due to the high cost of enzymes and their limited access to chitin. Thus, depolymerization of chitin is essential for optimal utilization by various microalgae and cyanobacteria in the manufacture of value-added products [145,146].

Sugarcane (*Saccharum officinarum*) is a crucial global crop, valued for its traditional role in sugar production and its growing use in the production of bioproducts and biofuels [147]. The principal objective of sugarcane cultivation has always been the production of sucrose, which underpins the sugar industry. Nonetheless, its biomass composition—comprising 40–50% cellulose, 25–30% hemicellulose, and 15–20% lignin—presents novel opportunities within the biorefinery framework. This facilitates the generation of high-value derivatives, including ethanol, succinic acid, xylitol, lactic acid, and other molecules that boost the bioeconomy [148]. Feedstocks containing sugars and starches are associated with the first-generation process, which uses edible biomass. Non-food lignocellulosic materials, such as corn stover and grasses, offer sustainable, potentially cost-effective alternatives for second-generation processes. This approach minimizes the competition between food and fuel while decreasing feedstock costs through efficient processing methods [149].

Organic waste streams serve as an environmentally friendly alternative to fossil-derived resources, as they do not directly compete with sustenance crops. Waste encompasses any organic material that is not the primary product for which the plants were initially cultivated (e.g., corn stover from maize), as well as any biomass-based by-product for which supply significantly surpasses need (e.g., glycerol from biodiesel) [150]. The organic component of solid waste can be converted into valuable products via microbial processes. Anaerobic digestion (AD) is an effective method for processing organic waste, particularly compared to alternative techniques such as thermal, biological, and chemical methods. It involves a sequence of biochemical reactions, including hydrolysis, acidogenesis, acetogenesis, and methanogenesis. AD is a more viable technology for treating organic waste and generating renewable energy than aerobic processes, as it requires lower energy input and produces less sludge. This approach reduces pollution by minimizing CO_2_ emissions while utilizing methane releases as possible fuel sources [151].

Glycerol is a multifaceted compound that serves as a feedstock for the production of several compounds. Glycerol, a by-product of biodiesel production, is readily available and cost-effective, making it an appealing precursor for chemical synthesis. The synthesis of alternative compounds from glycerol is crucial for reducing waste and generating new products from byproducts. The primary compounds that can be synthesized from glycerol include ethanol, butanol, hydrogen, glycerol carbonate (GC), PUFAs, animal feed, and propionic acid [152]. All thermochemical conversion methods have significant potential for large-scale use of glycerol as a feedstock. Before utilizing crude glycerol or other lignocellulosic biomass for bioenergy production, various steps, particularly pretreatment, significantly influence the ultimate yield, productivity, and product quality. The integration of biofuels into current energy systems is crucial to advancing smart, sustainable cities. Consequently, the output of crude glycerol generated by the biodiesel sector is poised for continued expansion [153,154].

Molasses is another feedstock of industrial significance. This byproduct of the sugar sector contains substantial quantities of readily accessible sugars and is available in liquid form, allowing direct use in fermentation. Additional benefits of molasses include its comparatively low cost and regional accessibility, with 88 sugar producers across the EU alone. Historically, the fermentation of molasses has been examined across many organisms and products, and it is extensively used for the synthesis of citric acid, alcohols, and SCPs [155,156]. The valorization of agro-industrial byproducts, including soy molasses, is consistent with the fundamentals of renewable bioeconomy and sustainability. Soy molasses contains a high concentration of raffinose family oligosaccharides (RFOs), including raffinose and stachyose, which possess prebiotic properties and are utilized in the food and nutraceutical sectors. Moreover, its increased carbohydrate content positions it as an attractive option for biofuel production, such as bioethanol and biogas, thereby expanding alternative energy options and reducing the investment required for the process, as it accounts for approximately 20% of the cost associated with conventional carbohydrate sources [157].

Lignocellulosic biomass (LCB) hydrolysates represent a potential alternative source of concentrated sugars for microbial lipid synthesis, contingent upon the tolerance of the microorganisms to inhibitory compounds produced during pretreatment processes. The inhibitory compounds consist of acetic acid, furfural, and 5-hydroxymethylfurfural (HMF). Research indicates that cultivating heterotrophic microorganisms on lignocellulosic biomass hydrolysates under nitrogen-limited conditions enhances lipid accumulation via the utilization of sugars derived from holocellulose. The generation of SCOs from LCB hydrolysates represents a viable biorefinery strategy for producing biodiesel, polymers, and biosurfactants [158,159].

Although traditional acid and alkaline lignocellulose pretreatments improve sugar release, they entail high chemical use, the production of inhibitory chemicals (such as furfural and HMF), and substantial wastewater treatment requirements, all of which increase the environmental burden. Although there are still issues with processing time and cost, more environmentally friendly options, such as biological pretreatment and green solvent-based techniques (e.g., ionic liquids), exhibit lower toxicity, less waste, and better recyclability [160,161]. Additionally, pretreatment has a major impact on the overall environmental footprint of lignocellulosic bioprocesses, particularly on energy consumption, greenhouse gas emissions, and chemical-related effects, according to current life-cycle assessment (LCA) research. These findings demonstrate that when included in biorefinery systems, green pretreatment technologies, particularly those that reduce chemical inputs and permit solvent recycling, offer superior environmental performance. Therefore, to ensure alignment with circular bioeconomy principles, the selection of pretreatment strategies for microbial lipid production should be guided not only by sugar yield but also by comprehensive sustainability criteria, such as LCA outcomes, process integration, and waste minimization [162,163].

Fundamentally, the physical attributes and chemical constituents of the biomass constitute the most critical determinants of a biomass source’s suitability for biofuel conversion. The standards for these quality criteria vary significantly between conversion procedures [164]. Variability in feedstock quality, including carbohydrate, ash, and moisture content, significantly influences the maximum theoretical product yield in biofuel conversion processes. Moreover, variability in biomass quality, characterized by low carbohydrate content and elevated ash levels, can substantially increase operational expenses and diminish overall plant efficiency, primarily by increasing downtime and equipment use during preprocessing. Reduced biomass carbohydrate levels and increased ash content adversely affect the theoretical ethanol yield during biofuel conversion, thereby increasing the amount of non-convertible materials. Biomass qualities, such as yield variability, can be significantly influenced by drought [165].

Acid pretreatments effectively degrade the lignocellulosic structure by disrupting the glycosidic bonds linking lignin to hemicelluloses. This approach solubilizes a significant proportion of hemicelluloses (often exceeding 90% by weight), while concurrently diminishing cellulose concentration and partially extracting lignin. Dilute-acid pretreatment solubilizes hemicellulose and lignin at temperatures between 160 and 220 °C and at acid concentrations of 0.05% to 5%. Response times may range from a few seconds to several minutes [166]. Alkaline chemicals are used in alkali pretreatment to extract lignin and some amount of hemicellulose from LCB. Alkali pretreatment reagents, such as sodium hydroxide, potassium hydroxide, and ammonium hydroxide, have demonstrated greater efficacy. This method enhances enzyme access to cellulose, thereby increasing saccharification efficiency. Alkali pretreatment alters lignocellulosic structure by enlarging cellulose, diminishing crystallinity, and lowering the degree of polymerization. This enhances the internal surface area while eliminating uronic acids and acetyl groups in hemicelluloses [167,168]. By applying enzymes to break down the intricate structure of lignocellulose, enzymatic pretreatment improves the accessibility of lignocellulosic biomass for subsequent conversion processes [169]. This method has the benefit of not producing any inhibitors or greenhouse gases that might interfere with the process. Microorganisms, particularly bacteria and fungi, are favored for commercial enzyme production owing to their capacity for large-scale cultivation. Laccase, a copper-dependent polyphenol oxidase, is a crucial enzyme in pretreatment degradation [170,171,172]. The strong structures of lignocellulosic biomass are disrupted by hydrothermal pretreatment, also known as liquid hot water pretreatment, which uses elevated water temperatures (120–260 °C) and steam explosion at 2–6 MPa. This method improves the enzyme accessibility and digestibility of biomass by efficiently hydrolyzing cellulose and disassembling hemicellulose and lignin. With increasing water temperature, hydrogen bonding strength diminishes, leading to a gradual increase in ionization. Hydrothermal treatment facilitates the degradation of glycosidic linkages in lignocellulosic biomass. Increased temperatures enhance water flow and promote mass transfer, thereby accelerating chemical reaction rates by decreasing liquid viscosity. Furthermore, hydrothermal pre-treatment improves the conversion of acetyl groups in hemicellulose to organic acids, thereby facilitating the depolymerization of polysaccharides [173,174].

## 5. Downstream Processing of Microbial Lipids

### 5.1. Cell Disruption Techniques

Cell disruption is a critical step in the downstream processing of intracellular biomolecules and serves as the initial separation step, aiming to achieve high yield with minimal losses and product contamination. Consequently, the extraction of intracellular biomolecules increases downstream costs, and selecting the optimal disruption approach is a significant challenge, particularly for large-scale operations [175,176]. Various techniques for facilitating microbial cell breakdown include severe, gentle, and combination procedures.

#### 5.1.1. Mechanical

Mechanical methods are considered severe due to their non-specificity and reliance on mechanical principles, which may degrade the wall and destroy its strength-providing components. These methods encompass high-pressure homogenization (HPH) and bead milling [175,177].

**Figure 5:**
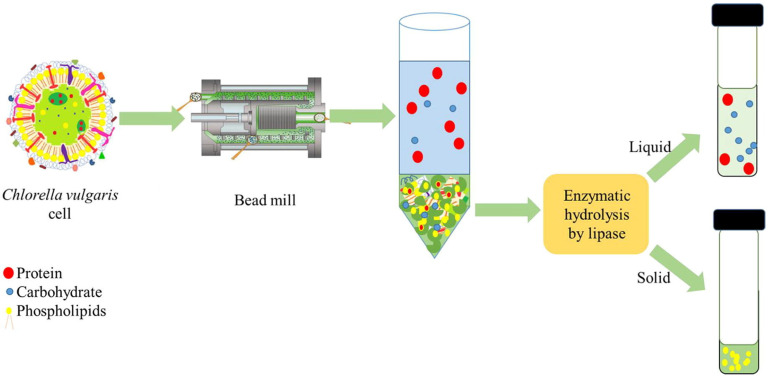
Schematic representation of the hybrid bead milling and enzymatic hydrolysis method. Reprinted from an open access source [182].

Bead milling, or bead homogenization, is a mechanically intricate procedure for cell disruption. This approach induces cell disruption through shear forces generated by cell rotation and by cell grinding between beads. Bead–cell collisions are allegedly involved in the mechanism through which bead milling destroys cells. Due to component variability across devices, such as the grinding chamber design or the agitator, discrepancies are likely when protocols are repeated across devices. Parameters such as bead dimensions, bead density, and bead loading need to be optimized based on the stiffness of the microorganism’s cell wall, the medium thickness, and the flow rate. The optimal biomass concentration for maximal cell disruption must be determined to improve overall process yield. The procedure’s parameters interact in complex ways, necessitating precise adjustments to ensure efficacy and efficiency. Insufficient settings may result in heightened energy usage, which can be substantially mitigated through optimization [178,179,180]. Bead milling is recognized as a promising method for disrupting the rigid cell walls of microalgae, with reports indicating that passing *Chlorella* cells through bead mills can yield over 90% disruption efficiency [181]. A hybrid bead milling and enzymatic hydrolysis method was established for the fractionation of the primary valuable biomass constituents from the microalga *C. vulgaris*. It showed that the best recovery yield for all components was achieved following the enzymatic hydrolysis of bead-milled biomass using lipase at 37 °C and pH 7.4 for 24 h, resulting in 88% lipids in the solid phase, while 74% carbohydrates and 68% proteins were extracted in the liquid phase (Figure 5) [182].

This method is highly effective for resilient fungal structures such as *Saccharomycescerevisiae*, *Yarrowia lipolytica*, and *Aspergillus* spp., offering advantages such as rapid DNA extraction for diagnostic purposes and effective release of bioactive compounds. However, parameters such as bead size, speed, and the number of passes must be optimized for specific fungi to maintain disruption effectiveness while preserving analyte integrity [183,184].

High-pressure homogenization (HPH) utilizes elevated pressure as the suspension traverses a nozzle at high velocity, resulting in a rapid pressure drop that, along with the opposing collision force from the seat valve and impact ring, induces mechanical disruption of the cells. The HPH can be implemented on a broad scale because it does not cause excessive heat dissipation, even when disrupting dense pastes. The nozzle orifice determines the effectiveness of cell disruption by a high-pressure homogenizer, the suspension’s velocity and viscosity, and the loading pressure at which the solution is pushed through the nozzle [185,186]. HPH leads to the generation of cellular debris, which may entail additional downstream costs for filtration and removal. The solutions must also be pumpable. The risk of agglomeration in the low-pressure chamber or small nozzle is significant, especially when the sample experiences temperature increases due to strong shear stress, and the equipment is typically expensive to maintain and operate [187]. The integrity of the microalgal cell wall significantly affects the disruption efficiency of HPH. Five distinct microalgae, specifically *Arthrospira platensis* PCC 8005, *Chlorella vulgaris* SAG 211-19, *Haematococcus pluvialis*, *Porphyridium centum* UTEX 161, and *Nannochloropsis oculata*, were assessed for the impact of cell wall structure on high-pressure homogenization efficiency. The cyanobacteria *A. platensis* exhibited the highest disruption efficiency with an 88% release of soluble proteins, whereas *H. pluvialis* demonstrated the lowest rupture efficiency with a 41% release of intracellular proteins [188]. In general, Gram-positive bacteria are more resilient to high-pressure homogenization than Gram-negative bacteria. Gram-negative bacteria have thinner cell membrane walls than Gram-positive bacteria, which has been linked to this effect. Yeast and fungus exhibited resistance to HPH, situated alongside Gram-positive and Gram-negative bacteria. Notwithstanding their thicker membrane compared to that of Gram-positive bacteria, their larger size and unique cell wall composition, rich in proteins, mannans, and glucans, reduced their resistance to HPH stress [189].

#### 5.1.2. Chemical/Solvent-Based

**Figure 6:**
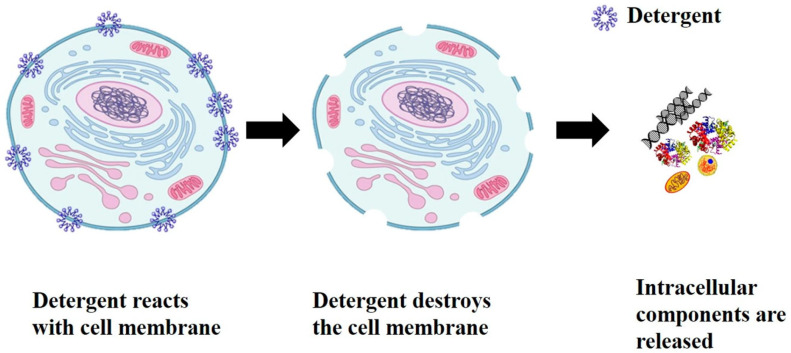
Cell lysis with detergent to permeabilize the cell membrane. Reprinted from an open-access source [190].

Chemical lysis techniques employ lysis buffers to rupture the cell membrane. Lysis buffers compromise the cell membrane by modifying the pH, destabilizing lipids and proteins, and increasing membrane permeability. Cell lysis buffers can use detergents to solubilize membrane proteins and rupture the cell membrane, releasing their contents. Figure 6 illustrates the cell lysis technique, in which a detergent is used for the chemical rupture of the membrane [190].

Organic solvents such as toluene, acetone, ethanol, dimethyl sulfoxide, chloroform, and ether are frequently employed for cell wall permeabilization to extract intracellular biomolecules, enzymes, and bio-pigments [175]. The method of cell disintegration by acid hydrolysis primarily aims to liberate bound lipids by breaking down lipid–starch and lipid–protein intermolecular interactions. Hydrochloric acid (HCl) processing has been employed as a conventional method for lipid extraction from various oleaginous yeasts [191]. Hydrolysis of HCl (1 M HCl, incubated at 60 °C for 2 h) was determined to be the most efficacious method for damaging the cell walls of both yeast strains, *Cyberlindnera jadinii* ATC9950 and *Rhodotorula glutanis* LOCKR13, when compared to osmotic shock, pasteurization, freezing/defrosting, and homogenization using zirconia balls (0.5 mm in diameter) [192]. Analogously, base treatment is often used to catalyze the breakdown of polysaccharides and proteins. In contrast to acid treatment, base treatment is significantly less successful and is therefore infrequently employed for lipid recuperation from oleaginous yeasts. Kruger et al. [193] found that the base treatment (NaOH) for lipid recovery from *Lipomyces starkeyi* was ineffective, yielding a recovery of less than 1%, whereas the acid treatment (H_2_SO_4_) recovered 91.5% of the intracellular lipids. The failure is probably due to the formation of a gel from the leftover particles, which led to inadequate agitation during the subsequent hexane extraction step. At elevated pH, gel formation may occur due to deprotonation or denaturation of cell walls, altering surface charges.

#### 5.1.3. Green Alternatives

Supercritical fluids possess distinctive physicochemical properties, including elevated densities and reduced viscosities, making them ideal extraction solvents. CO_2_ is the most advantageous supercritical fluid employed in extraction procedures. The non-flammability, non-toxicity, process selectivity, and simplicity of recovery of supercritical carbon dioxide (SC CO_2_) are its most significant attributes for utilization. SC CO_2_ is the most widely used supercritical fluid due to its ease of use and cost-effectiveness as a solvent, and is also considered a green solvent. SC CO_2_ treatment is an alternative technique for deactivating microorganisms. Increased pressure and temperature of SC CO_2_ correspond to a greater percentage of inactivated microorganisms. Increased pressure increases the solubility of CO_2_ in water, thereby facilitating the formation of carbonic acid. Elevated temperatures enhance the diffusion of CO_2_. The membrane’s fluidity improves, thereby enhancing the excretion of cellular substances [194,195]. In the response surface model for SC-CO_2_ extraction of *Mortierella alpina*, data indicated that pressure was the predominant factor influencing lipid recovery, with interaction effects observed between temperature and cosolvent ratio, as well as between pressure and cosolvent ratio. The lipid recovery from SC-CO_2_ extraction attained 92.86% at 201 bar, 58.9 °C, with a cosolvent ratio of 1:15 [196].

#### 5.1.4. Comparative and Techno-Economic Perspective

Although mechanical techniques (such as high-pressure homogenization and bead milling) are the most industrially scalable and often yield the highest disruption efficiencies (>90%), they are also energy-intensive and raise downstream processing costs. On the other hand, chemical/solvent-based techniques (such as hexane systems and chloroform–methanol) enable simultaneous disruption and high lipid recovery but also pose challenges related to solvent recovery, toxicity, and environmental impact. Supercritical CO_2_ and ionic liquids are examples of green alternatives that offer greater selectivity and lower environmental impact, but they are still limited by high capital costs and limited industrial maturity [197,198]. The Folch method and the Bligh and Dyer method, which rely on chloroform–methanol solvent systems to recover total lipids, are widely used as reference protocols among traditional laboratory-scale extraction techniques because of their high extraction efficiency and reproducibility. However, both are challenging to scale industrially because of solvent toxicity, large solvent volumes, and safety concerns. Similarly, Soxhlet extraction provides thorough lipid recovery and is commonly used for analytical purposes. However, despite its robustness, its industrial use is limited by long extraction times, high solvent consumption, and poor energy efficiency [199,200]. Overall, while mechanical methods remain the industrial benchmark, the high cost and energy requirements of downstream processing highlight the need for hybrid, scalable extraction strategies that balance efficiency with improved techno-economic and environmental performance.

### 5.2. Lipid Extraction Methods

The Folch method and the Bligh and Dyer method are regarded as the gold standards for lipid extraction. Although these procedures were originally designed for lipid extraction from animal tissues, the high efficacy of chloroform/methanol in isolating principal lipid classes has led to their widespread use for lipid extraction from various plant and animal samples (fluids and tissues). The Folch method is typically preferred for lipid extraction from solid tissue, while the Bligh and Dyer method is considered beneficial for biological fluids [199]. The Bligh and Dyer method employs a solvent mixture of chloroform, methanol, and water at a ratio of 1:2:0.25 (*v*/*v*/*v*). The monophasic ternary solution is transformed into a biphasic condition through dilution with supplementary chloroform and water. This solvent–water combination at this ratio resulted in a biphasic separation, with the lower chloroform phase containing all extracted lipids. Some researchers used 1 M NaCl instead of water to inhibit the adhesion of acidic lipids to denatured lipids [191,200,201]. The Folch method closely resembles the Bligh and Dyer technique, yet employs a distinct solvent ratio of chloroform:methanol (2:1 *v*/*v*) and incorporates a washing step with a specified quantity of salt solution. The combination of solvents and cellular material yields a biphasic separation, with pure lipids in the lower stage and organic solvents in the higher phase [191,202].

The Soxhlet method is a conventional technique employed for lipid extraction in food samples. Initially, the sample is dried, ground to a fine powder, and stored in a porous thimble. An organic solvent, commonly hexane, serves as an extractant for organic solutes like lipids. The apparatus comprises three main components: a flask, an extraction chamber, and a condenser. Upon heating the flask, the solvent evaporates and ascends to the condenser, where it is recondensed into liquid form and collected in the extraction chamber, which holds the sample. As the solvent traverses the sample, it removes the fats and transports them back to the flask. This extraction process generally spans several hours (6–24 h) [122,203]. Compared with a modified Bligh and Dyer method using a methanol–chloroform mixture (2:1 *v*/*v*), Soxhlet extraction with the same solvent combination over five cycles yielded inferior lipid extraction efficiency from R. glutanis, achieving 98% and 50% extraction efficiency, respectively. The drawback of prolonged operation time (typically approximately 8 h) associated with conventional Soxhlet extraction can be mitigated by integrating Soxhlet extraction with microwave heating, thereby reducing the operation time to under 1 h [191].

Ionic liquids (ILs) are organic salts with a large cation and, optionally, an organic or inorganic anion. ILs are often characterized as green solvents and exhibit numerous advantageous physicochemical properties, including low vapor pressure, limited flammability, high ionic conductivity, strong solvating capacity, and thermal and chemical stability. In recent years, ILs have been used as solvents for lipid extraction and for the extraction of biological products from microalgal biomass. ILs can solubilize cell wall polysaccharides by forming hydrogen bonds between cationic and anionic species [175,204]. Agostinho et al. [205] examined a microwave-assisted extraction method using a biocompatible ionic liquid (IL) for lipid recovery from *Chlorella vulgaris*. Traditional techniques (Soxhlet, Folch, and Bligh and Dyer) were initially assessed for benchmarking purposes. Optimizing microwave intensity, extraction duration, and the algae-to-ionic liquid mass ratio revealed that a 1:8 (*m*/*m*) ratio, applied for 5 min at 750 W during microwave pretreatment, yielded the maximum lipid yield, with 10.61 ± 0.39% of lipids extracted from the supernatant alone. The subsequent extraction of pretreated biomass with an eco-friendly solvent mixture (ethyl acetate/ethanol, 1:1 *v*/*v*) increased total lipid recovery to 14.29 ± 0.75%, exceeding that of Soxhlet extraction with chloroform/methanol (13.04 ± 0.16%). The ILs were effectively recovered (about 85%) and reused with no notable degradation in performance or their structural integrity. Another study demonstrates the efficiency of lipid extraction from magnetically isolated microalgae using ILs, with subsequent reuse of ILs and magnetic nanoparticles (MNPs). MNPs effectively separated microalgae from the aqueous phase, achieving a separation efficiency of 99%. The isolated microalgae/MNPs slurry was treated with an ionic liquid to disrupt the microalgae cell wall, thereby facilitating lipid exposure for effective hexane extraction. Lysed cells were combined with hexane for 2 h to facilitate lipid extraction. The extraction efficiency reached 99% with ILs/hexane, compared with only 5% with hexane alone [206].

## 6. Techno-Economic and Environmental Assessment

LCA facilitates the comparative evaluation of the life cycle of product systems. Modifications of LCA, including Life Cycle Costing (LCC) and process Techno-Economic Analysis (TEA), can be used at different phases of the life cycle to assess the most cost-effective approaches for raw material manufacturing, product and coproduct processing, or end-of-life management. These methodologies can complement an environmental LCA to assess technical, economic, and environmental concerns [69]. An LCA study of microbial oil systems identified key areas for improvement across environmental, economic, and social dimensions. The results of the LCA and LCC underscore the necessity for enhancements in heat consumption. Given that natural gas is used for steam for yeast lysis, investigating alternative, viable methods for oleaginous yeast lysis is imperative. Further enhancements may include the use of sustainable thermal sources, such as biogas derived from the valorization of sludges generated within the system. The S-LCA analysis indicates that enhancing plant production through improved efficiency and yields is the most effective way to mitigate sustainability hotspots. Enhanced sustainability performance may involve employing modified yeast strains that facilitate lipid extraction in milder conditions and yield significant quantities of extracellular lipids [207].

McKuin et al. [208] evaluated the life-cycle implications from “cradle to factory-gate” of fish oil, compared with whole-cell and extracted oils from *Schizochytrium* mixed with canola oil. The findings indicated that substituting fish oil with microalga *Schizochytrium* and canola oil have greater implications for environmental sustainability than previously recognized. The integration of *Schizochytrium* with canola oil will significantly reduce the need for marine biological resources; nonetheless, advancements in growing technology for heterotrophic microalgae are necessary to address environmental sustainability trade-offs. Addressing these trade-offs requires interdisciplinary advances across production domains, including the use of carbon from sugar feedstocks derived from sustainable waste products like plant cellulose, as well as improvements to sugar feedstock supply networks. In addition to environmental factors, substituting fish oil with *Schizochytrium* and canola oil could offer health advantages by eliminating heavy metal contamination and other pollutants of concern, while preserving the omega-3 fatty acids found in farmed fish flesh. Consequently, the trade-offs in environmental sustainability must be weighed against the human health benefits of using *Schizochytrium* as a substitute for fish oil.

Lipid extraction is considered a significant impediment to large-scale algae biodiesel production due to the use of toxic solvents and energy-intensive processes for cell destruction and solvent recovery [209]. Another study employed LCA to analyze the environmental impacts and primary energy demand (PED) of algae biodiesel manufacturing from “cradle to grave,” emphasizing alternative solvents for lipid extraction and identifying, for the first time, potential process design enhancements. Limonene, ethyl tert-butyl ether (ETBE), and cyclohexane are examined in conjunction with the reference solvent, hexane. The results indicate that the climate change (CC) and PED of algal biodiesel vary from 94 to 101 g CO2 eq./MJ and 1.59 to 1.65 MJ/MJ, respectively, with hexane exhibiting the lowest CC and PED, while limonene demonstrates the highest values. Lipid extraction is recognized as a primary challenge in downstream processing due to the significant energy expenditure associated with cell disruption (e.g., high-pressure homogenization) and solvent recovery. Employing an acid pretreatment for cellular disruption before lipid extraction decreases 11 of 18 environmental consequences and the PED of algal biodiesel across all solvents, including hexane [210].

The comparative LCA for several final algal products, including algal powder, bio-oil, total fatty acids, and residue recycling, remains constrained, particularly with autotrophic algal production for products beyond bio-oils and biofuels. Consequently, numerous autotrophic farmed microalgae have been selected for a comparative LCA among these species, along with a comprehensive step-by-step manufacturing process for *Chlorella* sp. The results indicated that optimizing algal species selection and final product choices, as well as algal cultivation methods, biomass separation, and drying processes, and choosing appropriate land and employing renewable electricity alongside nearby thermal power plants for CO_2_ or flue gas, could significantly reduce production costs and environmental impacts, thus highlighting the understanding of microalgal consumption choices considering contemporary global demands and constraints related to carbon sequestration [211]. Another study assessed the environmental and economic sustainability impacts of a production process for SCOs derived from the seaweed *Saccharina latissima*, which utilizes the oleaginous yeast *Metschnikowia pulcherrima*. Two different fermentation systems were evaluated, incorporating uncertainties related to seasonal fluctuations in seaweed carbohydrate output and fermentation efficacy into the analysis. From an environmental standpoint, the study suggests that seaweed-derived SCO lipids and fats can be compared with a terrestrial oil mixture, with a possible climate change impact estimated at 2.5–9.9 kg CO_2_ eq. kg^−1^ of refined SCOs. Notably, environmental impacts are predominantly influenced by energy demand during fermentation and upstream processing. From an economic standpoint, the cost of the seaweed feedstock significantly affected the oil’s break-even selling price [212].

Regarding biodiesel generation from vegetable oils, various crops have been examined, particularly jatropha and palm. The most significant factors in LCA were land use, where nitrogen fertilizers and crop yield are pivotal, and the extraction method. The primary consequence of the latter was attributed to energy consumption and solvent use in Soxhlet extraction, which may induce adverse carcinogenic and ecotoxicological effects. Waste cooking oil is pertinent at this point, as its integration with other novel methods, such as heterogeneous catalysis, is a suitable option for its valorization. Alternative raw resources, such as algae and animal fats, pose issues due to the low efficiency of oil extraction from these sources. Nonetheless, their application in biodiesel production is viable and holds significant promise, as it has been shown to reduce certain impact categories (for example, biodiesel production from microalgae often decreases greenhouse gas emissions due to CO_2_ sequestration) [213].

Although a substantial body of literature has applied TEA and LCA to biomanufacturing systems, existing studies indicate that most research remains segmented rather than comparative across configurations, substrates, and downstream schemes. TEA frameworks typically focus on process simulation and core economic metrics such as minimum selling price (MSP), capital expenditure (CAPEX), operating costs (OPEX), and profitability indicators like payback period or internal rate of return, but these are often reported for individual pathways in isolation rather than in a systematic horizontal comparison of alternative fermentation modes or feedstocks. Likewise, LCA research tends to concentrate on environmental impact categories within defined system boundaries rather than integrating these with TEA outcomes to contextualize economic feasibility limits or identify core cost bottlenecks across scales. As a result, existing analyses have not adequately clarified the economic thresholds for industrialization (e.g., target unit lipid cost, payback benchmarks) nor provided a structured comparative framework of key techno-economic parameters for different fermentation modes, feedstocks, and downstream processes that could guide investment decisions and scale-up strategies [214,215,216].

## 7. Outlook and Future Trends

The production of microbial lipids has advanced significantly over the past few years, driven by advances in strain evolution, metabolic engineering, and bioprocess optimization. Although laboratory-scale research has demonstrated remarkable lipid yields and product concentrations, commercial implementation is hindered by issues such as robustness, scalability, feedstock variability, and economic viability.

Simulations are essential for the development and optimization of fed-batch systems in microbial lipid synthesis. Numerous simulation studies exist, and it would be beneficial to implement them in practice to validate the assumptions. For instance, a computational framework utilizing dynamic flux balance assessment and small-scale metabolic models was established to assess and enhance lipid production in *Rhodosporidium toruloides* strains [217]. A separate initiative seeks to assess the simulation of biomass and oil synthesis with *Parachlorella* sp. through the ASPEN Plus software. The simulation relies on the fixation of CO_2_ by algae, which, through their cellular metabolism, convert it into oil, subsequently extracted and processed into biodiesel [218]. An evaluation of *Schwanniomyces occidentalis’s* lipid production capacity was conducted using modeling results and experimental data. The maximum experimental lipid production achieved was 0.43 glip/L h, with the lipid content in the biomass around 0.24 mass fraction. The biomass productivity has attained approximately 1.49 gx/L h, which is elevated for this type of cellular product [219].

In addition, next-generation biorefineries utilize a variety of feedstocks (such as algae, lignocellulose, and waste streams) and integrated processes to simultaneously extract multiple co-products (including proteins, pigments, lipids, chemicals, and fuels), advancing beyond single-product models to enhance economic viability and sustainability through circular economy principles, process integration, and the utilizations of microbial consortia for high-value products in food, pharmaceuticals, and materials. Essential tactics encompass consecutive extractions (e.g., water for proteins, solvents for lipids/pigments), leveraging by-products such as glycerol, and cultivating microbial platforms (algae, fungi) to optimize biomass valorization [220,221,222,223].

Developed strains must exhibit both phenotypic and genetic stability to be used in manufacturing, as they must sustain their intended phenotype over long periods under a variety of environmental conditions. Stability can be assessed by cultivating strains over multiple generations without selection pressure, followed by evaluating their efficiency under selective conditions. While few studies have performed short-term stability evaluations, comprehensive long-term assessments are infrequently conducted. Given their importance for industrial scalability, improved stability testing should be incorporated as a standard component of ALE protocols. This involves evaluating the stability of generated strains under industrially relevant conditions over multiple generations, ranging from tens to hundreds, depending on the organism and its application. Such datasets could enable the development of predictive machine learning models and are crucial for evaluating a strain’s suitability for industrial uses [93].

By significantly reducing experimental redundancy and increasing the precision of evolutionary predictions, the application of machine learning algorithms shifts from phenomenological observation to mathematical modeling. Technological developments are revolutionizing ALE execution. Automated hardware platforms and advanced bio-mutagenesis technologies have markedly accelerated adaptive evolution timescales. Concurrently, single-cell technologies, multi-omics, and high-throughput screening offer unmatched accuracy in tracking several evolutionary routes across concurrent investigations. These developments support the transition of ALE from system-level co-evolution to precise manufacturing at the protein scale by enabling comprehensive functional assessment of multi-site mutations, alongside advances in molecular biotechnology. The synergistic integration of ALE with rational design approaches has emerged as a viable approach for optimizing phenotypes, improving efficiency, and reducing costs [224].

The emergence of CRISPR-Cas systems was a significant advancement, facilitating precise targeted gene editing in microalgae. Microalgae, including diatoms, have the potential to use CRISPR technology to express specific genes and enhance industrial traits such as biomass yield and lipid accumulation. The use of genetically modified organisms (GMOs) to produce fourth-generation biofuel may mitigate the issues associated with the exhaustion of fossil fuels. Initial genetic access constraints were overcome by CRISPR applications, which primarily focused on gene knockouts and knock-ins using programmable nucleases (e.g., Cas9/Cas12). Nonetheless, just “cutting” DNA is fundamentally inadequate to address the complex issues of future-oriented metabolic engineering. Enhancing characteristics such as lipid production in excess, stress resilience, or intricate metabolic flux rerouting necessitates sophisticated, multi-faceted strategies: adjustable gene expression regulation, epigenetic modification, foundational adjustments, and dynamic management across multi-gene networks [225,226].

A primary impediment to SCO biorefineries achieving optimal outcomes is the limited availability of comprehensive technical studies, necessitating a technical–economic assessment of oleaginous bacteria for SCO production. The price of microbial oil was confirmed at USD 3000 per ton, comparable to cocoa butter, indicating huge potential for this raw material. Consequently, this technique not only enhances the availability of superior lipid raw materials of significant commercial value but also fosters the emergence of a new consumer market alongside innovative products derived from the newly created oils [227]. Future advancements in microbial lipid biomanufacturing will rely on enhanced integration of strain evolution, process modeling, and real-time process control, underpinned by data-driven and automated methodologies. Progress in synthetic biology, continuous processing, and the use of inexpensive, renewable feedstocks is anticipated to significantly improve the viability of large-scale microbial lipid production. Ultimately, closing the divide between laboratory innovation and commercial application will be essential for positioning microbial lipids as a viable and sustainable foundation for next-generation bio-based goods. Current oleaginous microorganisms can attain lipid levels of 40–70% of dry cell weight, with certain systems achieving almost 80%, illustrating significant biological potential for high-yield production [228]. Moreover, SCO production has attained greater flexibility regarding feedstock utilization, with organisms adept at converting lignocellulosic substrates, industrial waste streams, and volatile fatty acids (VFAs) into lipids, thereby remaining unaffected by climate and arable land limitations—an essential advantage over traditional plant oil production [4,19]. From an engineering standpoint, fed-batch methodologies have demonstrated a considerable improvement in lipid productivity, whereas continuous bioprocesses provide more consistent and scalable production frameworks. The incorporation of SCO synthesis into biorefinery frameworks, in which lipids are produced concurrently with other valuable products, enhances overall process economics and advances the technology toward practical application. The utilization of affordable alternative substrates is emerging as a focal point of study [3,228,229]. Ultimately, the convergence of advanced strain engineering, process intensification, and sustainable feedstock utilization is expected to drive microbial lipids toward becoming a competitive and scalable alternative to conventional lipid sources in the global bioeconomy.

## Figures and Tables

**Table 1:** Comparison of bioreactor configurations for microbial lipid production.

Bioreactor Configuration	Working Principle	Main Advantages	Main Disadvantages
Batch	Closed system with predetermined medium composition Substrates are gradually depleted while biomass, products, and by-products accumulate C/N ratio changes dynamically during cultivation.	Simple operation and control Valuable for determining optimal operating parameters (e.g., pH, aeration, agitation, media composition) No need for complex feeding strategies and control loops	Substrate depletion and by-product accumulation Poor control of the C/N ratio after initiation Risk of growth inhibition at high initial substrate concentration Lower lipid/substrate conversion efficiency Productivity losses due to downtime (cleaning, sterilization, regeneration)
Fed-batch	Nutrients supplied continuously or intermittently (bolus feeding) Substrate concentration is dynamically controlled C/N ratio can be regulated during cultivation.	Higher cell densities Better control of substrate concentration Improved C/N ratio regulation Avoids substrate inhibition Widely used in industry	Requires a feeding strategy design Risk of substrate limitation with constant feed rate Requires space for feed addition and possible volumetric increase during operation
Continuous	Constant input of fresh medium (C and N) and simultaneous removal of culture (bleed). Steady-state operation with constant cell concentration and substrate uptake rate Productivity is primarily determined by the dilution rate.	Maximized productivity Constant C/N ratio at steady state Allows continuous harvesting Reduced downtime Improved economic viability compared to batch bioreactors	Requires careful optimization of nitrogen level and dilution rate Higher contamination risk Risk of strain oil degradation Potential operational blockages Harder batch separation and traceability
Two-stage	Decoupled phases: Stage 1 (low C/N, nutrient-rich) for biomass growth; Stage 2 (high C/N, nitrogen limitation) for lipid accumulation May involve distinct substrates and/or cell recycling.	Optimized conditions for growth and lipid synthesis separately Reduced substrate consumption for excess biomass Higher lipid accumulation efficiency Better metabolic control of the lipogenic phase	Increased process complexity Higher viscosity at high cell density Reduced oxygen transfer Potential additional oxygen supply costs

## Data Availability

No new data were created.
